# Cardiac arrests in unmonitored wards: 3 years in a hospital

**DOI:** 10.1186/2197-425X-3-S1-A539

**Published:** 2015-10-01

**Authors:** A Iglesias Santiago, R de la Chica Ruiz-Ruano, A Sánchez González, M Colomo Gonzalez, L Navarro Guillamón, ARIAM-Andalucía

**Affiliations:** Virgen de las Nieves Hospital, Granada, Spain; Queen's Hospital, London, United Kingdom

## Objectives

To describe the characteristics of cardiac arrests (CA) occurred in unmonitored areas.

## Methods

It was performed a prospective cohort study, according to Utstein style, of every inhospital CA occurred in the universitary “Virgen de las Nieves” hospital during a period of 3 years (July 2009 to June 2012). CA in operating room, recovery and when extra-hospital resuscitation was initiated were excluded. All patients in whom resuscitation was not performed or in which it was suspended, because of the existence of an advance care plan, the existence of do not resuscitate orders or those in which resuscitation was deemed futile, were also excluded. It was also recorded the use of defibrillators (manual or automatic external defibrillator) before the arrival of the cardiopulmonary resuscitation team, regardless of whether shock was administered or not. We have analyzed and compared the arrests in monitored areas versus the rest. Results were expressed as percentages, means and medians ± standard deviation. Statistical analysis was performed using the chi^2^ test when the dependent variable (survival and shockable rhythm) was qualitative and the t-Student test when it was quantitative (interval CA-start of cardiopulmonary resuscitation).

## Results

During this period there were 297 patients suffering at least one CA episode. In hospital wards, 96 patients were resuscitated. The most common gender was male (61.5%) with a median age of 72 years (68.4 ± 12.8 years). About the location in unmonitored wards were CA occurred, the most frequent was the Internal Medicine ward, followed by the cardiology ward (16 and 12 patients). The most common etiology was respiratory (38.5%). Most of the initial rhythms were asystole (36.5%) and only 8.3% of them were shockable rhythms. Defibrillators (AED or manual) were placed only in 6 patients before the arrival of the CPR team. The median CA-arrival of the CPR team interval was 4 minutes (4.7 ± 3.8 minutes). Survival to hospital discharge was 15.6%. At discharge, 11 patients had good functional status, 2 had moderate disability and 2 severe disability.

Comparing hospital wards with the rest of the hospital, no statistically significant differences in overall survival or delay in defibrillation were found, but we found differences in the presence of less common initial shockable rhythms (chi^2^ = 9.7 p = 0.002) and greater interval CA-start of resuscitation maneuvers (t=-6.5; p < 0.001).

## Conclusions

Cardiac arrests occurred in hospital wards show shockable initial rhythms less frequently and later onset of cardiopulmonary resuscitation maneuvers than the rest of the hospital but similar hospital survival.

